# HIV Cure Research: Ethical and Real-World Practical Considerations for Pediatric and Adolescent Populations

**DOI:** 10.1093/jpids/piag003

**Published:** 2026-01-16

**Authors:** Elise Tirza Ohene-Kyei, Jessica Salzwedel, Karine Dubé, Yusuf H Wada, Mark Cotton, Deborah Persaud, Allison Agwu

**Affiliations:** Division of Pediatric Infectious Diseases, Department of Pediatrics, Johns Hopkins University School of Medicine, Baltimore, MD, United States; Division of Adolescent and Young Adult Medicine, Department of Pediatrics, Johns Hopkins University School of Medicine, Baltimore, MD, United States; University of Maryland, College Park, Department of Behavioral & Community Health, Baltimore, Maryland, United States; AIDS Vaccine Advocacy Coalition (AVAC), New York City, NY, United States; Pediatric AIDS Virus Elimination Collaboratory (PAVE), Baltimore, MD, United States; Division of Infectious Diseases and Global Public Health, University of California San Diego School of Medicine, San Diego, CA, United States; Pediatric AIDS Virus Elimination Collaboratory (PAVE), Baltimore, MD, United States; Society for Family Health, Department of Health Policy, Research, Advocacy and Influencing, Abuja, Nigeria; Pediatric AIDS Virus Elimination Collaboratory (PAVE), Baltimore, MD, United States; Family Center for Research with Ubuntu, Stellenbosch University, Cape Town, South Africa; Division of Pediatric Infectious Diseases, Department of Pediatrics, Johns Hopkins University School of Medicine, Baltimore, MD, United States; Pediatric AIDS Virus Elimination Collaboratory (PAVE), Baltimore, MD, United States; Division of Pediatric Infectious Diseases, Department of Pediatrics, Johns Hopkins University School of Medicine, Baltimore, MD, United States; Pediatric AIDS Virus Elimination Collaboratory (PAVE), Baltimore, MD, United States; Division of Infectious Diseases, Department of Medicine, Johns Hopkins University School of Medicine, Baltimore, MD, United States

**Keywords:** pediatrics, children, adolescents, HIV cure, HIV cure research, ethics, social, psychosocial

## INTRODUCTION

In 2024, an estimated 2.4 million children and adolescents under 19 years of age were living with human immunodeficiency virus (HIV)^1^ worldwide, with ~260 000 new diagnoses^1–4^ that year, mainly from perinatal HIV transmission. Nearly 89% of these young people reside in Sub-Saharan Africa^2^^,^^5^ and face a lifetime of HIV treatment and its multifaceted sequelae.^6^ Cure, the eradication of rebound-competent HIV with durable HIV control off antiretroviral treatment (ART), could transform HIV infection into a disease-free state, with minimal risk of onward transmission.^7^ Lifelong ART with its attendant toxicities may no longer be necessary. In principle, longitudinal comorbidities could be minimized, and a normal, healthy life span promoted if durable ART-free control or remission were achieved.^8^^,^^9^ Despite increased understanding of how HIV persists in its viral reservoirs and its barriers to sustained ART-free control or remission, HIV cure remains elusive. Notably, HIV has now been shown to be curable in adults through stem-cell transplantation for the treatment of malignancies, forming a scientific proof of concept for this therapeutic goal.^9^^,^^10^

Currently, achieving sustained viral suppression (VS) is the key initial step for considerations for HIV cure.^11^ Yet only 57% of children and adolescents with HIV are on ART vs. 77% of adults^1^ and pediatric VS lags behind adults (64% vs. 79%) one year after ART initiation.^12–14^ This disparity in VS underscores unique reasons to prioritize pediatric HIV cure research. HIV cure strategies in perinatal infection require unique considerations given knowledge of timing of exposure to HIV which requires very early testing and treatment. This strategy is under study in clinical trials and has provided the proof-of-concept for ART-free remission in children.^15^^,^^16^ Additional strategy under study in adults and not yet studied in children is the use of latency reversal agents in combination with immunotherapeutics, with the goal of re-awakening HIV from its state of viral latency to ultimately purge HIV reservoirs. Another key consideration for HIV cure research is the critical component of the need for analytical treatment interruption (ATI), where ART needs to be stopped, and participants carefully monitored for rebound viremia to ascertain the efficacy of interventions. Typically, rebound viremia occurs within 2-4 weeks of ART discontinuation, providing a metric to assess efficacy.^9^ Clinical reports have shown cases of ART-free virologic control or post-treatment control in early treated adults^17^^,^^18^ and children^19–21^ who self-interrupted ART (Table 1). Although cure research is progressing, the current mainstay of pediatric cure studies is very early ART with and without broadly neutralizing antibodies (bNAbs) (Table 3). Other immune interventions include HIV vaccines that are still in the planning phase.

**Table 1:** Published Cases of Sustained Virologic Control off ART in Pediatric and Adolescent HIV Cure Research

Case	Initial VL (copies/mL)	ART Initiation	ART Duration Before Interruption	Remission Duration
Mississippi Child^21^^,^^56^^,^^57^	19 812	30 hours	18 months	27 months
South African Child^19^^,^^57^	>750 000	2 months	10 months	14 years and ongoing^a^
Visconti Teenager^19^^,^^20^	2 170 000	3 months	5.8-6.8 years	15 years and ongoing^a^
P1115 children^57^				
Participant A	>5 million	1 day	5.6 years	80 weeks
Participant B	96	2 days	5.5 years	>60 weeks and ongoing^a^
Participant C	1969	1 day	5.3 years	>48 weeks and ongoing^a^
Participant D	87	2 days	5.7 years	>44 weeks and ongoing^a^
Participant E	118	1 day	5.4 years	8 weeks
Participant F	15 017	1 day	5.5 years	3 weeks

a Indicates cases where rebound has not occurred. These individuals may have undetectable viral loads using standard assays but detectable RNA and/or DNA using non-standard assays.

Abbreviations: ART, Antiretroviral Treatment/Therapy; HIV, Human Immunodeficiency Virus.

With increased focus on cure strategies, we should carefully delineate, anticipate, and prepare for the unanticipated implications of studying and achieving cure from all perspectives,^22^ given that HIV affects physical, emotional, and psychological health.^23^ These implications are different for children and adolescents, deserving of unique considerations appropriate for their ages and level of maturity.

Ethically, HIV cure research should have strong scientific premise, with attention to engagement of adolescent populations. Ethical research also requires appropriate protections, practical and thoughtful policies governing continued equitable research, access, eventual distribution of effective strategies, and consideration for curtailing exploitation.^24^ Lo and Grady proposed an eight-part framework for ethical HIV cure research emphasizing collaborative partnership, social value, scientific validity, fair participant selection, favorable risk–benefit ratio, independent review, informed consent, and respect for participants and communities.^25^ To date, most ethical considerations in HIV cure research have focused on adults.^26^^,^^27^ With increasing global pediatric research, these ethical considerations should be reviewed.

The Pediatric Adolescent Virus Elimination (PAVE) Martin Delaney Collaboratory is the first Collaboratory focused on the science and community perspectives of HIV eradication/control in children.^28^ Our multi-disciplinary group of biomedical scientists, community advocates, socio-behavioral scientists, and bioethicists used the Lo and Grady framework to explore ethical and practical considerations and real-world implications for pediatric and adolescent HIV cure research,^25^ including the rationale for including children and adolescents in these early phase studies, and their multifactorial life course considerations.

## DISCUSSION

### Rationale for Including Children and Adolescents in Cure Research

Children and adolescents <19 years-old are considered vulnerable populations in ethical guidance and regulatory frameworks, requiring special considerations when including them in HIV cure research.^29^ Some researchers and ethicists believe they should be included in cure research only after safe, effective strategies have been identified in adults. Even then, therapeutic trials of antiretrovirals occur in a stepwise fashion, starting with older adolescents before younger populations are studied, including neonates.^30^ This approach to studying ART has led to marked delays in children’s access to newer treatments by decades.^31^ To avoid these delays, it is imperative that we have a prospectively planned pediatric framework, analogous to those developed for ART in pregnancy (by the WHO/IMPAACT), and for tuberculosis treatment and prevention in pregnancy (by the WHO). These frameworks should define criteria and timelines from the outset, for sequentially including adolescents, children and neonates in studies of new therapeutics for HIV, including those aimed at ART-free control and cure.

**Table 2:** Multifactorial Life Course Considerations for HIV Cure Research in Infants, Children, and Adolescents with Early Acquired HIV

	0 – <2 Years	2 – <5 Years	5 – <12 Years	12 – 17 Years
	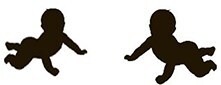	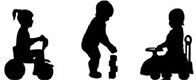	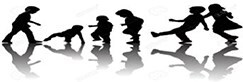	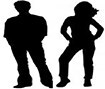
**BIOLOGICAL/PHYSICAL CHARACTERISTICS**				
**Biology (size, blood volume, reservoir size, pharmacokinetics (PK), enzyme maturity, kidney/liver function changes; biopsy/sampling capacity/ acceptability)**	Plasma protein binding of drugs typically decreased, though this remains drug-dependent. Limited blood volume for sampling, and biopsies are not encouraged, unless necessary. PK are variable by drugs; however, there is often increased activity of drug-metabolizing enzymes in this age group, yet function is generally lower, at about 20-70% the function of adults.^58^		PK are variable by drugs, with increased activity of drug-metabolizing enzymes in this age group. Enzyme function starts to near the function of adults.	Similar protein binding to adults. Blood sampling and biopsies are more acceptable but are also only encouraged when extremely necessary. Enzyme functions similar to adults and often differ by sex.
**Neurocognitive**	Brain rapidly developing and growing. Infant or child starts to develop basic language skills and emotional connections.	Improved language, communication skills, and ability to understand and use symbols (words and numbers). Child begins to develop a sense of self and the ability to consider the thoughts and feelings of others.	Refined cognitive, language, and social–emotional skills. Better at problem-solving, planning, and organizing thoughts and actions. Children are more independent and begin to understand and navigate the social world around them.	Significant brain development, especially in decision-making, impulse control, and social cognition. Adolescents continue to refine their cognitive, language, and social–emotional skills, and they become more aware of their own thoughts and emotions and how they relate to others.
**Sex/reproductive, hormones, and** **puberty**	Puberty has not set in, and sex and reproductive hormones are dormant.		Puberty begins in the latter part of the age range. Sexual and gender identity start evolving.	Sexual and gender identity have often developed, and sexual activity may commence.
**Mental health**	HIV-related stigma and discrimination experienced by the caregiver can contribute to developmental delays and negative mental health outcomeswhich may in turn affect the child’s well-being.	Cognitive dysfunction, learning disabilities, depression, anxiety, difficulty forming friendships and social connections, bullying, and rejection may lead to feelings of isolation, low self-esteem, and self-worth.	Prone to negative mental health outcomes such as depression and anxiety, due to stigma and discrimination from peers, family, etc. Behavioral problems and difficulty with self-regulation due to limited access to medical care and support services. Cognitive dysfunction and negative mental health outcomes may affect learning capacity, educational attainment, employment, etc. Difficulty accepting HIV status and negative impact on self-esteem and self-worth. Difficulty in school due to lack of understanding or discrimination. Challenges with adherence and taking daily oral ART	
**HIV CARE**				
**HIV management**	Caregiver	Caregiver	Caregiver à Self-management	Full transition to self-management.
**ART regimens**	Simpler regimens		Regimens may become more complex if nonadherence and resistance increase/develop.	Regimens may get more complex as the risk of resistance increases – both resistance to medication and resistance to taking medication. Further, available regimens may vary based on in-country and/or state regulations, caregiver insurance or available finances. Injectables become an option around this age group. Desires for long-acting formulations with lower dosing frequency may develop to reduce ART adherence challenges. This will affect the ability to join HIV cure-related trials with ATIs due to PK tails.
**ART Side Effects and Toxicity**	Side effects are often regimen-specific. Long-term effects have ranged from mitochondrial toxicity, anemia, granulocytopenia, neutropenia, hepatotoxicity, kidney bone disease, weight gain with lipodystrophy, ie, abnormal fat distribution, and lipoatrophy – fat loss,^59^^,^^60^ especially with older ART regimens. Many of these are rare with current regimens.			More ART options are available and tolerated in this population. There is however the possibility of drug interactions with illicit drug use, or hormonal contraceptives. Side effects and toxicities from earlier ages may tend to progress and worsen with time.^59^^,^^60^
**Care delivery**	Pediatric care	Pediatric care	Pediatric care	Pediatric and adolescent care and transition into adult care
**Adherence**	Adherence is dependent on caregiver involvement.	Adherence remains dependent on caregiver involvement.	Adherence variable; may wane with decreased caregiver involvement.	Adherence is mostly dependent on self. Possible resistance to taking HIV medications in adolescence.
**Disease progression**	Due to an immature immune system, infants and children may experience rapid progression of HIV disease if treatment is delayed. Children are also more susceptible to common childhood infections and opportunistic infections (OIs). However, upon treatment initiation, they often have good immunologic response to ART.			Lower levels of viral suppression, due to non-adherence, comorbidities, etc.
**PSYCHOSOCIAL**				
**Risk and behavioral factors**	Caregiver’s emotional well-being and ability to provide appropriate care for the infant or child. Stigma and discrimination experienced by the caregiver can affect the infant or child’s well-being. Caregiver’s physical well-being, habits, and behaviors can have an impact on the infant or child’s well-being.			Limited access to information and education, difficulty accepting HIV status, and disclosing status to others; stigma and discrimination; negative impact on self-esteem and self-worth, difficulty forming relationships and social connections; psychological distress, and negative mental health outcomes, risk-taking behaviors (eg, tobacco & substance use) may commence, poor health outcomes, lifestyle factors/modifiable risk factors; potential sexual debut.
**Disclosure**	Disclosure is unlikely. However, there may be unintentional disclosure, in care settings and/or as part of research. There could be unintended consequences when there is inadvertent disclosure to everyone else except the child in the trial.		Caregiver disclosure often begins around this age range. The possibility of unintentional disclosure remains.	Disclosure to others (friends, partners, etc.). The possibility of unintentional disclosure remains. Conversations about onward disclosure and U=U, etc. as sexual activity may commence, preparing for increased self-efficacy toward transition to adulthood, etc.
**Informed consent and assent**	Cannot provide informed consent.	Cannot provide informed consent.	May be able to provide some form of assent; however, caregiver consent sometimes operationally can supersede it in real life. Assent should trump parental consent and a child’s refusal to participate in a study should be respected	Often able to provide informed consent and/or assent depending on factors such as jurisdiction and unique situation.
**Autonomy**	The child is unlikely to have autonomy in any situation.			While there may be a semblance of autonomy, caregiver(s)’ wishes may overrule.
**Stigma**	Caregiver stigma is often present, often affecting the child. Unlikely to experience personal stigma at this age.		May begin to experience stigma both internal and external.	Likely to experience both internal stigma and external stigma. May also experience increased intersectional stigma due to race, ethnicity, sex, gender, socio-economic status (SES), etc.

Abbreviations: AIDS, Acquired Immune Deficiency Syndrome; ART, Antiretroviral Treatment; HIV, Human Immunodeficiency Virus; NIH, National Institutes of Health; OI, opportunistic infections; PK, Pharmacokinetics.

Regulatory – approvals for older kids may be included with that in older adults.

Adapted from Adolescents and young adults with early acquired HIV infection in the United States: unique challenges in treatment and secondary prevention.^38^

**Table 3:** Age-Specific Considerations for HIV Cure/Interventions/Studies

	0 – <2 Years	2 Years – <5 years	5 – <12 Years	12-17 Years
	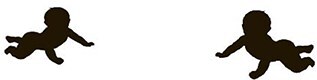	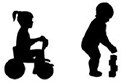	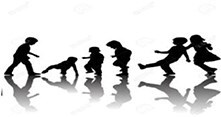	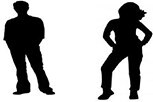
**A.VERY EARLY ART INITIATION – ART initiation in the first few hours – days of life.**	This intervention is primarily present in these age groups, to reduce HIV reservoirs, and thus help achieve remission, by reducing reservoir size.^51^ This strategy has shown the most promise in pediatric populations, such as in the Mississippi and South African cases – Table 1.)	This intervention is not applicable in later ages or life stages.		
**ANALYTICAL TREATMENT INTERRUPTION (ATI)**	ATI is currently not recommended in younger populations under the age of two. There is often rebound viremia by week four of treatment interruption.^33^	Older children who often face ART adherence challenges are likely to have difficulty with ART re-initiation post ATI.		
**IMMUNE-BASED APPROACH – Immune-based approaches are more likely to have a greater impact in much younger pediatric populations where viral diversity is lower than in older populations.**	**C.i. Broadly Neutralizing Antibodies (bNAbs)** –**Target regions of HIV, to cause virus neutralization by forming antigen–antibody complexes that promote immune clearance; may enhance HIV-specific immune responses**			
	Can maintain viral suppression in children after very early ART treatment.^51^			In older adolescents, similar to adults, bNAbs show some promise; however, resistance remains a limitation, hence further studies on dual/triple combinations of bNAbs as well as long-term bNAbs with fc effectors, and other strategies that make them more potent are underway.
	**C.ii. Stem Cell Transplantation –Eliminating the host immune system, and replacing in vivo immune cells with a donor who has natural immunity to CCR5-tropic HIV.** (Not currently under study in pediatric populations)			
	This approach is NOT being pursued for commercial use however is being optimized for individuals with blood cancer and HIV. It has successfully produced cures, all in adults. Stem cell transplantation has multiple challenges, such as graft vs. host disease (GVHD), which could be even more devastating in children.			
	**C.iii. Gene & Cell Editing – Altering *in* or *ex vivo* immune cells to create resistance against HIV, increase identification or clearing potential of immune cells toward HIV**			
	This strategy is also under investigation in cancer and sickle cell for both adult and pediatric populations and has shown some promise. Differences between pediatric and adult immune systems may allow for increased durability of some vector-based approaches.			
	**C.iv. Latency Reversal - Inducing HIV-1 expression to expose latently infected cells for immune clearance – Uses latency-reversing agents (LRAs) to reactivate latently infected cells, inducing HIV-1 gene transcription, resulting in clearance of infected cells either by viral cytopathic effect, or immune cell recognition and clearing of infected cells.**			
	This strategy has been studied only in adults and has not shown a very significant reduction in the size of the replication or rebound-competent HIV reservoir. Further research is underway, in combining this strategy with immunotherapies, to show better promise.^51^			
	**C.v. Permanent Silencing - Silencing the expression of HIV-1 for prolonged drug-free control.**			
	This emerging intervention may be more successful in younger populations who have very small viral reservoirs.^61^ However it has not yet been studied in these populations.		In older adolescents, this may need to be combined with other HIV cure strategies such as gene editing and conserved vaccines, HIVconsvX & HTI which are in the planning phase. These require safety & immunogenicity.	

Abbreviations: AIDS, Acquired Immune Deficiency Syndrome; ART, Antiretroviral Treatment; NIH, National Institutes of Health; OI, opportunistic infections; PK, Pharmacokinetics.

**Table 4:** Current Pediatric Cohorts Exploring HIV Cure-Related Research (Adapted from Advances in Pediatric HIV-1 Cure Therapies and Reservoir Assays^51^^,^^62^)

Planned and Currently Occurring HIV Cure Research In 2021, the National Institutes of Health (NIH) awarded approximately $US 53 million in annual funding over the next 5 years to 10 research organizations in a continued effort to find a cure for HIV. Only one grant of $US 5.7 million (10.8%) is focused specifically on HIV cure research in infants and children – the Pediatric Adolescent Virus Elimination (PAVE) Martin Delaney Collaboratory at the Johns Hopkins University, Baltimore.		
Trial	Description	Location
EIT (Early Infant HIV Treatment)	Very Early ART with the goal of drug-free remission	Botswana
HIV-Netherlands Australia, Thailand Research Collaboration	Early ART with the goal of confirming the safety and effects of prime-boost vaccine regimens.	Thailand
Pediatric HIV/AIDS Cohort Study (PHACS)	Longitudinal studies of reservoirs and inflammation among those with longstanding viral suppression (Influence of age at virologic control on peripheral blood HIV reservoir size and serostatus in perinatally exposed or infected people.	United States
Johns Hopkins Cohort – Long-term Pediatric Reservoir Cohort Study	Immune activation and exhaustion marker expression on T-cell subsets in adolescents treated with ART and young adults with perinatal HIV-1 infection as correlates of viral persistence	United States
Children with HIV Early Antiretroviral (CHER) Cohort	Children between 7 and 12 weeks with HIV at birth were randomized to receive ART at the time of diagnosis or when CD4 count had decreased to less than 20%. The cohort is now adolescents/young adults and are still in follow-up.	South Africa
IMPAACT P1115- Very Early Intensive Treatment of Infants Living with HIV to Achieve HIV Remission (NCT02140255)	The study explores the effects of early intensive antiretroviral therapy (ART) with or without a broadly neutralizing antibody (bNAb) on achieving HIV remission (HIV RNA below the limit of detection of the assay) among infants living with HIV.	United States, Argentina, Brazil, South Africa, Malawi, Kenya, Uganda, Tanzania, Zambia, Zimbabwe, Haiti, Thailand
IMPAACT 2039	Phase 1 study of HIVconsvX vaccine regimen and broadly neutralizing antibodies	United States

Abbreviations: AIDS, Acquired Immune Deficiency Syndrome; HIV, Human Immunodeficiency Virus.

### Biological Differences and the Potential for Early Intervention to Avert Disease Progression and Potentiate ART-Free Remission and Cure

Children have less developed immune systems when HIV infection is established, with differing quality and quantity of immune responses from adults.^32^ They experience rapid HIV progression with delayed ART initiation, and experience initial plasma viremia up to 10 times greater than that in adults, which is sustained for much longer, without ART.^32^^,^^33^ Their underdeveloped immune systems also make them more susceptible to opportunistic infections (OI).^34^ There is also evidence that after age two, children and adolescents may be better vaccine candidates than adults, with more naive T-cells & less immuno-senescence, and thus better antibody response to vaccines.^35^

Early ART initiation is lifesaving, preventing disease progression and increasing survival, and therefore a global standard of care.^32^^,^^34^ A unique biomarker of early effective ART from infancy is illustrated by the maintenance of HIV sero-reversion and seronegative antibody status through later childhood and adolescence with effective sustained VS, often detected as between 12 and 24 months of life.^36^ This HIV seronegative state could yield insightful scientific clues toward sustained virologic control. Paradoxically, absence of HIV antibodies may increase the risk for acute retroviral syndrome (ARS) when HIV rebounds after ATI.^37^

### Impacts of Long-Term ART and HIV Care

Life-long ART adherence is physically, mentally, and economically challenging. Newer ART regimens are better tolerated compared to earlier options, though drug toxicity eg, cardiometabolic toxicities, still remain a concern with life-long ART (Table 2). Pill fatigue and ART adherence challenges reduce ART effectiveness, leading to drug resistance.^38^ Children and adolescents with HIV have higher rates of depression and anxiety than their peers without HIV, due to internal and external stigma, disclosure dynamics, social rejection, discrimination, and isolation (Table 2).^39^^,^^40^ For younger children, caregiver dynamics such as guilt and stress may be challenging. HIV cure could ease this burden and improve caregiver-child dynamics by reducing daily ART-related stress.^41^^,^^42^ However, the frequent monitoring and risk of viral rebound could exacerbate stress, anxiety, and guilt.

**Table 5:** Ethical Considerations and Practical Unresolved Open Questions for Pediatric and Adolescent HIV Cure Research using the Lo and Grady Framework

***Collaborative Partnerships***	***Critical Questions***
Collaborative partnerships include community engagement, industry involvement, and multidisciplinary collaborations.^63^^,^^64^ Early community engagement should be responsive to evolving community demands.^65^ Several key guidance documents^66^ have limited representation from Global South communities and researchers focused specifically on pediatric and adolescent populations. More work must be done to expand community representation at the earliest stages of product development, particularly perspectives of adolescents and caregivers, from the Global South who can bring real-world expertise to conversations about product and protocol design and feasibility. With early collaboration, involving more researchers from the Global South specifically involved in pediatric and adolescent work, we can reach diverse representation and improve the chances of reaching cure. Further, partnerships between academics, civil society, funders, implementers, industry, and regulatory entities are vital for influencing funding and the regulatory environment for research. While research is emerging on adult needs and acceptance of potential cure strategies,^67^ perspectives of adolescents, children, and their caregivers from diverse contexts warrant further exploration.	*Should diverse stakeholders focus on pediatric HIV cure research be incorporated into ongoing conversations around cure guidance and target product profiles (TPPs) or do they need a parallel process?* *How do we manage expectations of potential participants, caregivers, and communities around pediatric HIV cure research?* *Who must be considered as part of the community? Which communities need to be included?*
***Social Value***	***Critical Questions***
Sustained virological control would benefit children and adolescents socially and economically by reducing clinic visits, missed school and unemployment in adolescence and young adulthood. Further, stigma increases anxiety and depression, reducing ART adherence, and potentially increasing substance and alcohol use.^68^Cure could reduce stigma and improve self-esteem within families, potentially minimizing the negative social effects.^41^ Current HIV cure and control strategies being pursued, based on biological characteristics, mechanisms of action, targets, and side effects, differ by age group (Tables 1 and 3). Ethical considerations for each intervention by age group must occur in consultation with participants and affected families. Geographic considerations such as accessibility of care centers and medication will also affect social value of different approaches in young people. Further, it is extremely important to consider the potential scalability of interventions and downstream access to curative approaches. Studies must be relevant to local priorities, aligned with available resources and healthcare systems, complementing existing efforts to prevent and treat HIV early. Relevant ethical questions must be addressed when implementing these studies. Considerations include future access and underlying public and global health impacts.	*What are some of the ethical considerations of specific interventions being pursued for different age groups?* *How do these interventions affect any social and economic structures?* *What does post-trial access or care look like based on HIV cure interventions?*
***Scientific Validity***	***Critical Questions***
Pediatric cases of cure or control provide strong scientific validity to pursue this research (Table 1). Currently, ATIs are not recommended for children under two years-old.^66^ Four of six toddlers, started on very early ART (within 48 h of life) without evidence of residual HIV, who have undergone ATI after two years-old in IMPAACT P1115 did not rebound after ATI.^52^ Age should be considered when assessing questions of scientific validity. Newborns have maternal immunity via maternal antibodies.^69^ As maternal immunity wanes, infants are vulnerable until their immune systems mature, boosted by infections and vaccinations. Young children have active thymuses and more naïve T-cells than adults, hence a greater ability to restore CD4 and CD8 counts.^70^ Adolescents have robust immune systems with less susceptibility to new infections and do not have diseases of aging.^71^ Pediatric cure research interventions should be evaluated with age considerations – these are not one-size-fits-all. Several products are under investigation in adults to delay time to rebound,^62^ but few are being investigated in children because of current physical and technological limitations of pediatric populations.^72^ There remain uncertainties around appropriate age cut-offs for this type of research. Adequate recommendations are needed to determine which interventions should proceed. These include *efficacy* – the ability of the intervention to suppress HIV completely and prevent viral rebound or disease progression off ART, *safety* – with emphasis on potential side effects and toxicity, *feasibility* – cost, accessibility, ease of administration, and *effect on the quality of life* – impact on the child’s ability to attend school, engage in physical activity, and have normal life and life expectancy. Interventions appropriate for older adolescents based on their physiologic traits may need additional considerations in younger adolescents or children. Ethical guidance has traditionally recommended that clinical trials start in older populations and sequentially move to younger populations.^24^ This guidance should be reconsidered as some interventions may show increased efficacy in younger populations. For example, naturally occurring bNAbs develop faster in children than in adults.^72^^,^^73^ In examining the ethical considerations of HIV cure interventions by specific age populations, we must carefully assess suitability, potential benefits, appropriate timing for pediatric ATIs, and how we can accurately translate adult research into pediatric populations.	*What are some trial implementation issues to be considered to ensure scientific validity (*eg*, blood volumes, diagnostic issues, monitoring, adherence, ancillary care, and follow-up)?* *Which HIV cure interventions should be tested in pediatric populations to enhance scientific validity?* *Is there a scientific rationale for different interventions to be pursued depending on age?* *When is it reasonable to test a single intervention versus combination interventions in pediatric populations?* *When is it reasonable to conduct ATI trials in pediatric populations?* *Are there scenarios where research on valid scientific questions should not be conducted because the value added is not significant enough for pediatric populations?* *Are there ways to accurately translate cure from adult research into pediatrics?*
***Fair Selection of Participants***	***Critical Questions***
Many factors influence fair participant selection. A study of 281 mother–child pairs in South Africa suggested that male children have unique immune factors that allow for more durable HIV suppression,^74^ unlike in adults where females frequently show better control off therapy than males.^75^ Geographical location, along with biological sex, may affect HIV cure strategies due to variations in clades, microbiomes and ART access across regions.^76^ Additional research is required on how different clades and innate immune systems might influence HIV cure protocols. Biological factors (Table 2) may also affect protocol inclusion and exclusion criteria and should be considered in discussions of fair participant selection. Enrollment should balance clinical concerns, community needs and access challenges particularly in resource-limited settings, as ATI trials require intensive monitoring, frequent visits, and sufficient laboratory infrastructure, often limiting participants to those near research sites or with highly motivated caregivers. In brief, questions around fair participants selection to be considered, include biological factors, eligibility for pediatric studies, and ensuring equitable consideration of study populations, beyond proximity to research facilities.	*How should we set inclusion and exclusion criteria in different age populations?* *Who would be eligible for observational versus interventional HIV cure trials and for ATIs?* *What are possible recruitment strategies for pediatric HIV cure trials?* *How do we prevent inadvertent disclosure of HIV in the conduct of pediatric and adolescent clinical research?* ^77^ *Does geography affect the risk in people with HIV (PWH)? To what extent should it be considered?* *How do we ensure inclusion across geographical regions? To what extent should it be considered?* *How is access to strategies made equitable across locales? How will post-trial access be made equitable across locales?*
***Favorable Risk/Benefit***	***Critical Questions***
Pediatric populations are diverse. Given the potential physical and psychosocial benefits of curing HIV at an early age, inclusion of early age groups in parallel with other pediatric and adult populations should be considered. There should be evidence of immune-based control over the risk of potential for increased reservoir seeding during rebound viremia with ATIs. These risks must be minimized and be reasonable relative to potential benefits. Beyond potential clinical risks, we must consider potential social, mental health, and financial risks, for pediatric groups, including stigma (psychosocial risk), loss of future trial eligibility (opportunity risk), and identity shifts and changes in social interactions. Adam Castillejo – The London Patient, cured of HIV via homozygous CCR5 Δ32 stem cell transplantation from a naturally immune donor,^78^ faced negative reactions and community distancing after going public.^79^ No pediatric cure cases have become public; however, pediatric survivors link HIV to their identities, raising concerns about how cure may alter self-perception and social interactions.^80^ When considering the potential social benefits of stigma reduction among older adolescents, psychosocial benefits must be balanced with unknown harms from loss of community. For them, community loss may include losing both peer networks, and established provider relationships, as care moves from infectious disease providers to general pediatric providers. The ultimate judgment on acceptable benefits to risks is complex because some risks, particularly for pediatric populations, are unknown.^81^ Pediatric HIV cure research presents a unique challenge: early phase trials may not provide direct clinical benefits, and researchers must report this.^26^ More research is needed to determine acceptability of interventions and understand how ATIs can affect young participants and their caregivers at different ages. To assess benefits/risks, pediatric participation guidance in ATI trials, with stakeholder perspectives is needed. Guidance should vary by age and will have different implications in young adults, particularly those who have only known U=U and have had fewer opportunities to disclose their HIV status.^12^ Ethicists must also weigh the complex potential positive and negative effects of disclosure. Would including sexually active adolescents warrant disclosure of sexual activity, exposing them to repercussions from caregivers, and/or partners? Guidance should also include community input on risk definitions over time at individual and community levels.	*What are the ethical considerations around conducting research in pediatric populations where the direct benefits to the participant do not outweigh the benefits to the scientific community and general population of pediatric HIV cure research?* *How does HIV cure research participation shift the social interactions of participants?* *Do the planned studies avoid untoward risk (*e.g.*, altering dosing), particularly with agents/strategies with serious toxicities in other populations (human and non-human) or biologics with a long half-life in the body?*^22^^,^^23^ *What perceptions of risks do different age groups in different geographic populations have of HIV cure research?* *What are the definitions and expectations of HIV cure in different populations with perinatal HIV?* *How do we balance the long-term potential consequences of viral rebound in younger populations when the impact will likely be physiologic and/or neurocognitive?* *What are the ethical considerations for including sexually active adolescents in clinical trials involving ATIs?* ^82–84^
***Informed Consent/Assent***	***Critical Questions***
In pediatric studies, informed consent and assent may be challenging for all involved, due to evolving medical, regulatory, and public expectations. Assent is required for children over seven and all adolescent participants. Are there situations where assent can be given without disclosure? Although their assent carries less weight than adult consent,^85^ it signals respect for their autonomy, and informs caregivers of their options and values. Challenges may arise when caregivers’ (legally accepted representatives) and adolescents’ wishes differ. For example, parental guilt over perinatal HIV may pressure children too young to assent to research.^41^ In such cases, caregivers must understand their decision-making limits in the best interest of their child. Local IRB/ERC policies may also influence the consent and assent process and documentation.^86^ It should be noted that a child’s assent trumps parental consent and if a child refuses to participate in a study, they should not be included. Ethically and morally, a child’s right to agree to research should be respected. Informed consent should be transparent and simple while also reducing the risk of therapeutic misconception and misestimation.	*How much should caregivers and participants be told about the results of the research to mitigate therapeutic misconception or misestimation?* *How do we reduce the risk of therapeutic (or curative) misconceptions in pediatric HIV research?* *How do we reduce the risk of therapeutic misestimation in pediatric HIV cure research?* *What is the burden of consent during labor?* ^87^ *How to simplify the informed consent process for pediatric HIV cure research?* *What is owed to caregivers whose children are enrolled in pediatric HIV cure research?* *What is owed to children/adolescents who are enrolled in pediatric HIV cure research?* *What are the ethical considerations of conducting cure research before disclosure of diagnosis?*
***Independent Review***	
Independent non-biased review in research refers to objective evaluation by an external group.^25^ Local institutional review boards (IRB)/ethics review committees (ERC)’s approval should be obtained for clear, consistent guidelines with local authority that enhance research integrity. In multi-site studies across different settings, evidence-based approaches, sensitive to sociocultural norms should be developed to align with existing country-specific and international guidelines. Additional review may be required for decisions around ATIs. For example, the IMPAACT P1115 trial used an external review panel to determine the need for ATIs at specific timepoints. Similar panels may be valuable, particularly in early phase research, to evaluate participants’ suitability for ATIs. Beyond local IRB/ERC review, external expertise on specific topics (eg, ATIs) may be needed, along with strengthened regulatory capacity for innovative pediatric HIV cure research, particularly in resource-constrained settings.	*How can IRB/ERCs support HIV cure research in pediatric populations?* *How will these IRBs/ERCs be trained adequately in resource-limited settings?*
***Respect for Participants and Communities***	
Respect for participants and communities, highlighted in the Belmont Report, is fundamental to ethical human research.^25^^,^^88^ Transparent frameworks involving caregivers with clear decision points with opportunities for discussion, feedback, and shared decision-making are essential. Community members/stakeholders should be involved in research even at the protocol development stage, especially for research such as cure research which may be controversial and poorly understood. It is imperative to invest in community-related activities including developing research literacy materials, aiding in socio-economic empowerment, and promoting advocacy. Community networks provide accountability and are independent safeguards to build more open, inclusive, and diverse research systems. Caregivers have a pivotal role in following research schedules, participant retention, and sharing information. They must be fairly compensated for their time and participation, and their concerns (eg, costs, travel to sites, potential toxicities) addressed. Respect for participants, families, and communities is essential in pediatric HIV cure research.	*How can we ethically involve community networks in pediatric HIV cure research?* *What supports are needed to help community members provide input into pediatric HIV cure research protocols?* *How do we manage participant and community expectations around pediatric HIV cure research?*

Abbreviations: ATI, Analytical Treatment Interruption; ERC, Ethics Review Committee; IRB, Institutional Review Board; HIV, Human Immunodeficiency Virus.

### Psychosocial and Behavioral Implications of HIV

Young people have unique lifelong experiences ranging from identity formation, bullying, and peer pressure to the positive and negative effects of social media and transition to adulthood.^43^ These situations are also influenced by age of disclosure and awareness of their HIV status, which may or may not precede the development of their own perceptions of HIV, affecting their self-acceptance and internalized stigma. HIV cure implies freedom from fear or worry of transmission, disclosure and stigma, isolation and for some individuals, from poverty often perpetuated by HIV.^44^ Social determinants such as poverty, food and housing insecurity, and lack of healthcare are associated with HIV acquisition risk and poor outcomes in people with HIV.^45^ Challenges also arise in access to education, healthcare, housing, and other essential services.^38^ Key programs (eg, housing) have been developed to address these challenges for people with HIV. Unknown repercussions of “cure” in this population could include potential loss of employment (for adolescents/guardians), social services/resources, or medical care tied to one’s HIV status.^46^^,^^47^For adolescents entering sexual debut, ATIs can complicate the Undetectable = Untransmittable (U=U)^48^ era when undetectable HIV means no sexual transmission. Viral rebound may cause inadvertent disclosure to partners, stigma, and potential transmission. ATIs may increase stigma in asking young people to risk becoming viremic to show intervention efficacy.^49^ The possibility of becoming viremic may force behavioral change (eg, new onset condom use), increasing disclosure risk and potential physical or psychosocial harm.^50^

### Research on Cure Strategies in Pediatrics

The current mainstay of pediatric cure strategies has been very early ART initiation within the first two to seven days of life, with close interrogation for absence of evidence for a latent reservoir followed by ATI, usually around 3-5 years of age.^16^^,^^51^^,^^52^ Other studies are examining Immunotherapeutic as strategies to achieve sustained ART-free control. For example, the Tatelo study in Botswana showed that switching ART to two bNAbs led to 24 weeks of sustained control in a subset of children treated very early.^51^ Pediatric cure strategies being investigated in vitro examine whether innate immune-enhancing agents, such as toll-like receptor (TLR) agonists, can reactivate and purge latent HIV from the reservoir. Gene editing strategies are being explored in adult populations (Table 4). Studies of the latent reservoir size and characteristics and the immune profiles of adults with lifelong perinatal HIV help to inform cure research. Further, innovations for pediatric assays, such as improving diagnostics with minimal blood volumes, (eg, home-based blood collection for viral load testing that would only require capillary blood draws without venous access) are under development and can benefit cure research for all populations.^53^

## LO AND GRADY FRAMEWORK FOR HIV CURE RESEARCH AND OPEN QUESTIONS

The Lo and Grady framework^25^ provides guidance in assessing adherence to ethical principles. We review how this framework applies to pediatric HIV cure research, highlighting potential considerations and relevant open questions (Table 5).

## CURRENT STATE OF PEDIATRIC HIV FUNDING^54^^,^^55^

The provision of HIV care has led to the expansion of clinical support infrastructure globally, with programs such as the Ryan White Program and the President’s Emergency Plan for AIDS Relief (PEPFAR), domestically and internationally, respectively, which require ongoing congressional support. The dismantling of key treatment programs globally, is undermining VS and progress toward cure, while simultaneously underscoring the urgency and rationale for advancing HIV cure research. ^46^^,^^47^

In early 2025, abrupt funding cuts, targeting programs under the United States Agency for International Development (USAID) and PEPFAR, led to a sudden halt in many HIV initiatives worldwide.^54^^,^^55^ These cuts could effectively impede progress made toward pediatric HIV cure by undermining and dismantling pediatric HIV cure research initiatives. Beyond pausing research trials and delaying future innovations, sharp declines in testing and follow-up, particularly for children, reduced social support services for vulnerable populations, and reductions in the health workforce have decimated the infrastructure for effective implementation of ART distribution for prevention and treatment, viral load monitoring, and delivery of care. With these disruptions, the infrastructure and the first critical steps for pediatric cure research, engagement, testing, and ART delivery, are obviated. Progress toward pediatric cure cannot happen without strong advocacy to prioritize programs that support HIV care and treatment for all, especially children.

## CONCLUSIONS

Pediatric HIV cure research is crucial for eliminating pediatric HIV, averting a lifetime of ART, reducing morbidity and mortality, and improving overall quality of life. Given the complexity of pediatric HIV, a one-size-fits-all approach to cure research is unfeasible. This research should be conducted in parallel with adult research, and interventions should be evaluated individually,, taking into account specific needs and characteristics of young participants. Ethical considerations governing this research must be carefully examined, as research toward HIV cure in pediatric populations expands and innovates. Ethical questions must be prioritized along with biomedical and psychosocial research, even if the answers are complex. Importantly, the community is a vital partner whose input must be sought, respected, and incorporated early and repeatedly to ensure best practices and outcomes toward pediatric HIV cure. Lastly, funding for pediatric cure research must be prioritized, shielded from political shifts, toward the grand aim of HIV elimination in children.

## Data Availability

This manuscript is a review paper, and as such, does not include original data. The materials and information discussed are available through the original sources cited in the references.
